# Curcumin inhibits colorectal cancer progression by targeting PTBP1 and CDK2-mediated pathways

**DOI:** 10.3389/fonc.2025.1566889

**Published:** 2025-05-21

**Authors:** Hao Zheng, Shenglong Li, Ye Wang, Shuang Su, Yiheng Wang, Fujing Wang

**Affiliations:** Department of General Surgery Ward No.10, Second Affiliated Hospital of Harbin Medical University, Harbin, Heilongjiang, China

**Keywords:** Curcumin, colorectal cancer, PTBP1, CDK2, autophagy the effects of curcumin on crc cell viability, proliferation, migration, invasion

## Abstract

**Background:**

Colorectal cancer (CRC) remains a significant cause of cancer-related mortality worldwide. Curcumin, a natural polyphenol, has shown promise in targeting key cancer pathways, but its precise molecular mechanisms in CRC are not fully understood. This study investigates the anti-cancer mechanisms of curcumin on CRC progression, focusing on PTBP1 and CDK2 as critical regulators.

**Methods:**

The expression of PTBP1 was assessed in clinical CRC samples and curcumin-treated cells via PCR and Western blot. Functional assays—including CCK8, colony formation, flow cytometry, Transwell migration/invasion, and apoptosis/autophagy staining—were conducted to evaluate curcumin’s effects. CDK2 was identified as a direct target using pull-down, kinase activity, and immunoprecipitation assays. CDK2 knockout models were used to validate curcumin’s effects *in vitro* and *in vivo*.

**Results:**

Curcumin markedly downregulated PTBP1 expression, and suppressed CRC cell proliferation, migration, and invasion while promoting apoptosis and autophagy. Mechanistic analysis revealed direct inhibition of CDK2 by curcumin, disrupting the CDK2–c-MYC–PTBP1 regulatory axis. CDK2 knockout mimicked curcumin’s effects but reduced the cells’ sensitivity to the treatment. *In vivo*, curcumin significantly inhibited tumor growth and activated autophagy-related pathways.

**Conclusions:**

This study uncovers a novel mechanism in which curcumin suppresses CRC progression by targeting the CDK2–c-MYC–PTBP1 axis. These findings provide compelling evidence for curcumin’s therapeutic potential and support further clinical investigation.

## Introduction

Colorectal cancer (CRC) is a leading cause of cancer-related deaths worldwide (1). Despite advances in early diagnosis and treatment, the prognosis for advanced CRC remains poor (2–4). Tumor progression is driven by aberrant cellular proliferation, enhanced migratory and invasion (5, 6), and resistance to apoptosis (7, 8), underscoring the urgent need to identify effective molecular targets to improve treatment outcomes.

Curcumin, a natural polyphenol derived from Curcuma longa, has garnered considerable attention for its multi-faceted anti-cancer properties (9–11). It has been shown to inhibit tumor proliferation, induce apoptosis, and regulate autophagy across various cancer types. However, its precise molecular targets and mechanistic pathways in CRC remain poorly defined, thereby constraining its translational potential in clinical settings (12–14).

Polypyrimidine tract-binding protein 1 (PTBP1) is an RNA-binding protein involved in the regulation of alternative splicing, mRNA stability, and translation. Recent studies have implicated PTBP1 in tumorigenesis through its effects on cell proliferation and survival. Elevated PTBP1 expression has been observed in multiple cancers, including CRC, where it promotes tumor growth and metastasis (15, 16). Cyclin-dependent kinase 2 (CDK2), a key regulator of G1–S phase transition, is frequently dysregulated in malignancies and has been identified as a therapeutic target in CRC (17). In addition, CDK2 may regulate PTBP1 expression via phosphorylation of c-MYC, suggesting a potential CDK2–c-MYC–PTBP1 signaling axis in colorectal cancer (18, 19).

In this study, we investigated the anti-tumor mechanisms of curcumin in CRC and identified PTBP1 and CDK2 as critical downstream targets. Through a combination of bioinformatics analysis, *in vitro* functional assays, and *in vivo* xenograft models, we reveal a novel regulatory mechanism in which curcumin inhibits CDK2 activity, subsequently disrupting the CDK2–c-MYC–PTBP1 axis. These findings provide new mechanistic insights into curcumin’s role in CRC and highlight its potential as a targeted therapeutic agent.

## Methods

### Clinical sample collection

CRC tissues and paired adjacent normal tissues were collected from patients undergoing surgery at the Second Affiliated Hospital of Harbin Medical University. Ethical approval was obtained from the Medical Ethics Committee of the Second Affiliated Hospital of Harbin Medical University (Approval No. YJSKY2024-451), and informed consent was acquired from all participants. All tissue samples were snap-frozen in liquid nitrogen and stored at -80°C for further analysis.

### Bioinformatics analysis

Transcriptomic data from the GSE229613 dataset was analyzed to assess the transcriptional response of HCT116 cells to curcumin treatment. Potential molecular targets of curcumin were predicted using the PharmMapper database. The 3D structure (mol2 format) of curcumin was retrieved from the Traditional Chinese Medicine Systems Pharmacology (TCMSP) database.

### Cell culture and treatment

Human colorectal cancer cell lines CaCo2 and HCT116 were obtained from the Cell Bank of the Chinese Academy of Sciences, and cultured in Dulbecco’s Modified Eagle Medium (DMEM) supplemented with 10% fetal bovine serum (FBS), 100 U/mL penicillin, and 100 μg/mL streptomycin at 37°C in a humidified incubator with 5% CO_2_. Curcumin (≥98% purity; Source Leaf Biotech, Cat# S19245-5g) was dissolved in dimethyl sulfoxide (DMSO) to prepare a stock solution and diluted in DMEM to achieve final concentrations ranging from 0 to 200 μM. The final DMSO concentration did not exceed 0.1% in any group.

### CCK-8 assay

Cell viability was assessed using the Cell Counting Kit-8 (CCK-8; Beyotime Biotechnology). Cells were seeded into 96-well plates at 5,000 cells per well and incubated overnight. CCK-8 reagent (10 μL) was added to each well and incubated for 4 hours at 37°C. Absorbance was measured at 450 nm using a microplate reader.

### Colony formation assay

Cells were digested into single-cell suspensions using 0.25% trypsin and seeded into 35 mm dishes at a density of 1,000 cells per dish. Cells were incubated at 37°C with 5% CO_2_ for 10–14 days, and the medium was replaced every three days. Once visible colonies formed, cells were gently washed with phosphate-buffered saline (PBS), fixed with 4% paraformaldehyde for 30 minutes, and stained with crystal violet for 20 minutes at room temperature. Excess dye was removed with PBS. Images were captured, and colony numbers were quantified using ImageJ software.

### Cell line construction

Lentiviral particles carrying the target gene constructs were synthesized and purchased from Jiman Biotechnology Co., Ltd. (Shanghai, China). Cells were seeded into 24-well or 6-well plates and cultured until approximately 70% confluency. Lentiviral infection was performed in the presence of 8 μg/mL Polybrene (Beyotime, China) to enhance transduction efficiency. After 24 hours, the medium was replaced with fresh complete medium. Transduced cells were selected using puromycin (2 μg/mL) for 3–5 days, and stable cell lines were validated by fluorescence microscopy.

### Western blot analysis

Total protein was extracted using RIPA lysis buffer (Sangon Biotech) supplemented with protease and phosphatase inhibitors. Protein concentration was determined using a BCA Protein Assay Kit (Beyotime Biotechnology). Equal amounts of protein were separated by SDS-PAGE and transferred onto polyvinylidene difluoride (PVDF) membranes. Membranes were blocked with 5% non-fat milk in Tris-buffered saline with 0.1% Tween-20 (TBST) for 1 hour and incubated overnight at 4°C with primary antibodies against PTBP1, CDK2, c-MYC, Atg10, Beclin-1, LC3, P62, and GAPDH (ABclonal). After washing, the membranes were incubated with HRP-conjugated secondary antibodies and visualized using enhanced chemiluminescence (ECL; Heyuan Liji Biotech).

### Real-time PCR analysis

Total RNA was extracted using TRIzol reagent (BBI Life Sciences) according to the manufacturer’s protocol. cDNA synthesis was performed using the BeyoRT™ II cDNA Synthesis Kit (Beyotime Biotechnology) with genomic DNA eraser. Quantitative PCR was carried out using the BeyoFast™ SYBR Green qPCR Mix (2X, Beyotime Biotechnology) on a real-time PCR system. Relative gene expression levels were calculated using the 2^(-ΔΔCt) method, with GAPDH as the internal reference.

### Chromatin immunoprecipitation-qPCR

ChIP assays were performed using the SimpleChIP^®^ Enzymatic Chromatin IP Kit (Cell Signaling Technology) to assess c-MYC binding to the PTBP1 promoter. Cells were crosslinked with 1% formaldehyde, lysed, and sonicated to shear chromatin into 200–500 bp fragments. Immunoprecipitation was conducted using anti-c-MYC antibody (Abcam), with IgG as a negative control. After reverse crosslinking and DNA purification, qPCR was performed to quantify enrichment. Input DNA was used for normalization.

### Wound healing assay

Cell migration was assessed using a wound healing assay. Cells were seeded in 6-well plates to near confluency. A linear scratch was made using a sterile pipette tip, ollowed by PBS washing and curcumin treatment in serum-free medium. Wound closure was monitored at designated time points and imaged under an inverted microscope. Migration was quantified using ImageJ.

### Transwell invasion assay

Cell invasion was assessed using Matrigel-coated Transwell chambers (8 µm pore size, BD Biosciences). Cells were resuspended in serum-free DMEM and seeded into the upper chamber. The lower chamber was filled with DMEM containing 10% FBS as a chemoattractant. After 24 hours, non-invading cells were gently removed using a cotton swab, and the invaded cells were fixed with 4% paraformaldehyde, stained with 0.1% crystal violet, and counted under a microscope. Quantification was based on three randomly selected fields per insert.

### Acridine orange staining

Autophagy was evaluated using acridine orange (AO) and ethidium bromide (EB) double staining. Stock solutions of AO and EB were diluted in PBS to prepare working solutions with final concentrations of 1–5 μg/mL. Cells were incubated with AO-EB staining solution at 37°C for 2–10 minutes. The staining solution was then removed, and the cells were gently washed twice with PBS for approximately 10 seconds per wash to remove excess dye. Fresh culture medium was added to the cells before observation under a fluorescence microscope. Autophagic vesicles were identified based on fluorescence characteristics.

### Hoechst staining for apoptosis detection

Apoptosis was assessed using Hoechst 33342 staining (Beyotime Biotechnology). Cells were cultured on coverslips in 24-well plates and treated with curcumin at the desired concentrations. Following treatment, cells were fixed with 4% paraformaldehyde for 15 minutes at room temperature, washed twice with PBS, and incubated with Hoechst staining solution according to the manufacturer’s instructions. After staining, cells were observed under a fluorescence microscope to identify apoptotic nuclei, which were identified by chromatin condensation and nuclear fragmentation.

### Cell cycle analysis

Cell cycle distribution was analyzed using flow cytometry. Cells were harvested and centrifuged at 350 × g for 5 minutes to remove the supernatant. The cell pellet was resuspended in PBS and centrifuged again under the same conditions. The resulting cell pellet was gently dispersed, and while vortexing, 3–5 mL of pre-cooled 70% ethanol was slowly added dropwise to the sample. The cells were fixed at 4°C overnight. Following fixation, cells were centrifuged at 350 × g for 10 minutes, and the supernatant was discarded. The cell pellet was washed twice with 3–5 mL of pre-cooled PBS. After the final wash, the pellet was resuspended in PBS, and the cell count was determined. A total of 1 × 10^6^ cells were collected by centrifugation (350 × g for 5 minutes), and the supernatant was carefully discarded. The cells were resuspended in 0.5 mL of staining solution, mixed thoroughly by vortexing. After 30 minutes of incubation at 37°C in the dark, samples were analyzed by flow cytometry to determine phase distribution (G1, S, G2/M).

### LC3-mitotracker colocalization assay

Cells were seeded in 6-well plates and grown to 40–60% confluency. The culture medium was replaced with serum-free and antibiotic-free DMEM. For transfection, 125 μL of OPTI-MEM medium (serum- and antibiotic-free) was added to two microcentrifuge tubes. Plasmid DNA was added to one tube and gently mixed, while 1 μL of Lipo8000 transfection reagent was added to the other tube, avoiding contact with the tube walls. Both mixtures were incubated at room temperature for 5 minutes. The Lipo8000 mixture was then added dropwise to the plasmid DNA mixture, gently mixed, and incubated at room temperature for 10–15 minutes to form DNA-lipid complexes. The transfection complex (250 μL) was added dropwise to each well, ensuring even distribution across the well surface. The final transfection volume was adjusted to 1 mL per well. Cells were incubated at 37°C in a 5% CO2 humidified incubator. After transfection, the medium was discarded, and pre-warmed Mitotracker Red (MTR) staining solution was added to each well. Cells were incubated for 20 minutes at 37°C, followed by replacement of the staining solution with fresh buffer. Colocalization of LC3 and Mitotracker was observed and imaged under a fluorescence microscope.

### RNA immunoprecipitation assay

RIP was performed using the RIP Kit (Geneseed Biotech) according to the manufacturer’s protocol. Cells were crosslinked to preserve RNA-protein interactions and lysed to extract cellular components. The nuclear fraction was isolated, and chromatin was enzymatically digested to generate RNA-protein complexes. The concentration of chromatin and the efficiency of enzymatic digestion were assessed before proceeding. The RNA-protein complexes were immunoprecipitated using specific antibodies and Protein G magnetic beads. The immunoprecipitated complexes were eluted from the beads, and crosslinks were reversed to release RNA. RNA was purified using spin columns provided in the kit. The enrichment of specific transcripts was quantified by qPCR.

### CDK2 kinase activity assay

*In vitro* CDK2 kinase activity was measured using recombinant CDK2 and human c-MYC protein. The following reagents were prepared: 1× kinase assay buffer, radiolabeled ATP, recombinant human c-MYC protein, and recombinant CDK2. Cell extracts were prepared and mixed with recombinant human c-MYC protein, recombinant CDK2, varying concentrations of curcumin (0, 65, 100 μM or 0, 75, 125 μM), radiolabeled ATP, and 1× kinase assay buffer. The reaction mixture was incubated at 30°C for 30 minutes. The reaction was terminated by adding 6× loading buffer. All components of the reaction mixture were collected and subjected to SDS-PAGE followed by Western blot analysis. The results were visualized and analyzed after development.

### Pull-down assay

Curcumin-conjugated agarose 4B beads were prepared as follows: agarose beads were activated in distilled water and incubated overnight with coupling buffer (0.1 M NaHCO3, pH 12.0, containing 40% v/v DMSO) and periplogenin. Residual active groups were blocked using 1 M ethanolamine (pH 8.0) at 45°C. Beads were then washed three times with alternating pH buffer to remove unbound molecules. For protein binding, recombinant CDK2 protein was incubated with the prepared beads at 4°C overnight. After incubation, the beads were washed three times with RIPA buffer (150 mM NaCl, 1% NP-40, 50 mM Tris-HCl, 0.1% SDS, 1 mM EDTA, 0.25% sodium deoxycholate) to remove non-specifically bound proteins. The bound proteins were eluted using sample buffer and analyzed by Western blotting to detect the pulled-down CDK2 protein.

### Xenograft tumor model in nude mice

Xenograft models were established using 28 five-week-old male BALB/c nude mice. Colorectal cancer cells (2 × 10^6^ HCT116 or CDK2 knockout HCT116 cells) were subcutaneously injected into the flanks of the mice. The mice were randomly assigned to four groups (n=7 per group): (1) Control group (HCT116 + vehicle), (2) Curcumin group (HCT116 + curcumin), (3) CDK2 KO group (CDK2 KO HCT116 + vehicle), and (4) CDK2 KO + Curcumin group (CDK2 KO HCT116 + curcumin). Curcumin was administered by daily gavage at a dose of 100 mg/kg. At the end of the experiment, the mice were euthanized by cervical dislocation, and tumors were carefully excised. Throughout the treatment period, no visible signs of systemic toxicity—such as abnormal weight loss, lethargy, or abnormal behavior—were observed in the curcumin-treated group, suggesting that the administered dose was well tolerated. This study was approved by the Medical Ethics Committee of the Second Affiliated Hospital of Harbin Medical University (Approval No. YJSDW2024-244).

### Hematoxylin and eosin staining

Tumor tissues were dehydrated through a graded ethanol series (85% for 2 hours, 95% for 2 hours, 100% I for 1 hour, 100% II for 1 hour). Dehydrated tissues were then cleared by immersing them sequentially in xylene I, II, and III for 10 minutes each. The tissues were embedded in paraffin using three paraffin baths (40 minutes per bath) and sectioned into thin slices. Sections were deparaffinized and stained with hematoxylin, followed by differentiation in acid ethanol and bluing in running tap water. Sections were counterstained with eosin, dehydrated through graded alcohols, cleared with xylene, and mounted with coverslips. Tissue morphology was evaluated under a light microscope.

### Immunohistochemistry for Ki67

Paraffin-embedded tumor tissue sections were deparaffinized in xylene and rehydrated through a graded ethanol series. Antigen retrieval was performed by heating the sections in a citrate buffer (pH 6.0) at 95°C for 10 minutes. After cooling, sections were incubated with 3% hydrogen peroxide for 10 minutes to block endogenous peroxidase activity. The sections were then blocked with 5% BSA for 30 minutes at room temperature and incubated overnight at 4°C with a primary antibody against Ki67. After washing, sections were incubated with a secondary antibody conjugated to HRP for 1 hour at room temperature. Color development was achieved using DAB, and sections were counterstained with hematoxylin. After dehydration and clearing, coverslips were mounted, and staining was evaluated under a light microscope. Ki67-positive nuclei were quantified under a light microscope.

### Data analysis

All experiments were conducted in triplicate, and the results are expressed as mean ± standard deviation (SD). Statistical analyses were performed using GraphPad Prism software (version 10.1.2). Differences between groups were assessed using Student’s t-test for two-group comparisons or one-way ANOVA for multiple group comparisons. Data normality was assessed using the Shapiro–Wilk test prior to applying parametric analyses. A p-value < 0.05 was considered statistically significant. Graphical data were generated using GraphPad. For quantification of protein bands or staining intensity, ImageJ software was used.

## Results

PTBP1 expression is elevated in colorectal cancer and reduced by curcumin.

**Figure 1:**
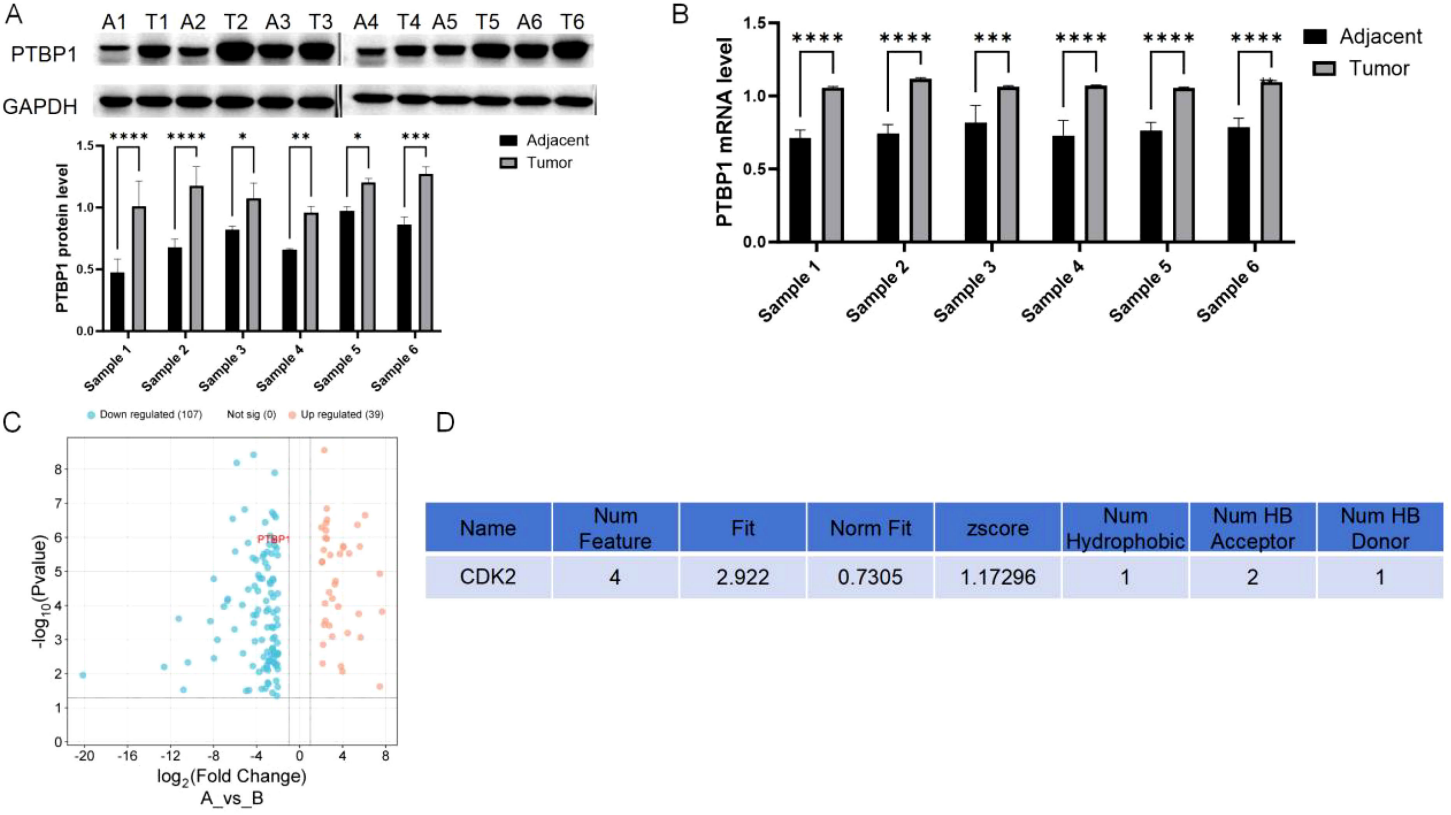
PTBP1 expression is elevated in colorectal cancer and reduced by curcumin. **(A)** Western blot analysis confirming the elevated expression of PTBP1 in CRC tissues. Representative images are shown. **(B)** PCR analysis shows that PTBP1 is significantly overexpressed in colorectal cancer tissues compared to adjacent normal tissues. **(C)** RNA sequencing data from GSE229613 indicates that curcumin treatment significantly reduces PTBP1 expression in colorectal cancer cells. **(D)** Target prediction analysis identifies CDK2 as a potential target of curcumin. Note: ns p > 0.05, * p < 0.05, ** p < 0.01, *** p < 0.001, **** p < 0.0001.

PCR analysis showed that PTBP1 was significantly overexpressed in colorectal cancer tissues compared to adjacent normal tissues, a finding confirmed by Western blot analysis (**Figures 1A, B**). This suggests a potential role for PTBP1 in colorectal cancer progression. RNA sequencing data from GSE229613 indicated that curcumin treatment markedly reduced PTBP1 expression in colorectal cancer cells, though the underlying mechanism remains unknown (**Figure 1C**). Target prediction analysis identified CDK2 as a possible molecular target of curcumin (**Figure 1D**).

Curcumin inhibits colorectal cancer proliferation, migration, and invasion while inducing apoptosis and autophagy.

**Figure 2:**
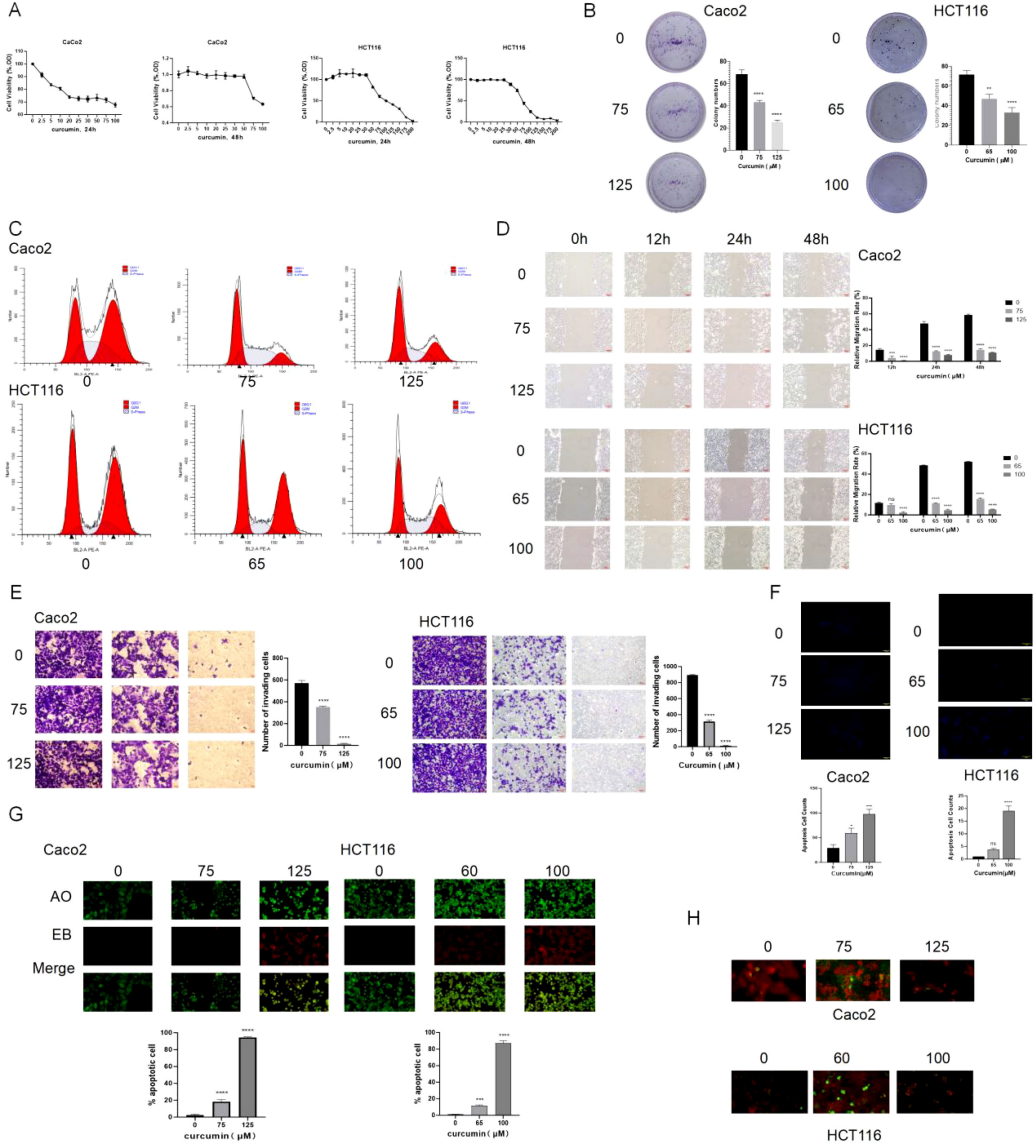
Curcumin inhibits colorectal cancer proliferation, migration, and invasion while inducing apoptosis and autophagy. **(A)** CCK8 assay shows that curcumin significantly reduces cell viability in both CaCo2 and HCT116 cell lines in a concentration- and time-dependent manner. **(B)** Colony formation assays demonstrate that curcumin treatment significantly inhibits the proliferation of CaCo2 and HCT116 cells. **(C)** Flow cytometry analysis reveals that curcumin induces G1 phase arrest in both cell lines, with a reduction in the S and G2/M phase populations. **(D)** Wound healing assay shows that curcumin significantly impairs the migratory ability of CaCo2 and HCT116 cells. **(E)** Transwell invasion assay indicates that curcumin treatment suppresses the invasive capacity of both cell lines. **(F)** Hoechst staining shows increased apoptosis in CaCo2 and HCT116 cells following curcumin treatment. **(G)** AO staining reveals enhanced autophagic activity in curcumin-treated cells. **(H)** LC3-mitochondria colocalization assays suggest that curcumin treatment promotes autophagy in both cell lines. ns, p > 0.05; *p < 0.05; **p < 0.01; ***p < 0.001; ****p < 0.0001. Data are presented as mean ± SD, n = 3 for each experiment.

Cell viability assays using CCK8 showed that curcumin reduced the viability of CaCo2 and HCT116 cells in a concentration- and time-dependent manner, with the two cell lines exhibiting different sensitivities to the treatment (**Figure 2A**). Based on these observations, subsequent experiments employed curcumin concentrations of 75 μM and 125 μM for CaCo2 cells, and 65 μM and 100 μM for HCT116 cells. Colony formation assays revealed that curcumin significantly suppressed the proliferation of colorectal cancer cells compared to untreated controls (**Figure 2B**). Flow cytometry analysis indicated that curcumin induced G1 phase arrest, accompanied by a reduction in S and G2/M phase populations (**Figure 2C**). Transwell invasion and wound healing assays demonstrated that curcumin treatment impaired the migratory and invasive capabilities of colorectal cancer cells (**Figures 2D, E**). Apoptosis analysis revealed a significant increase in apoptotic rates in cells treated with curcumin (**Figure 2F**). Curcumin also enhanced autophagic activity, as evidenced by acridine orange staining and LC3-mitochondria colocalization (**Figure 2G, H**).

Curcumin modulates key molecular targets in colorectal cancer cells.

**Figure 3:**
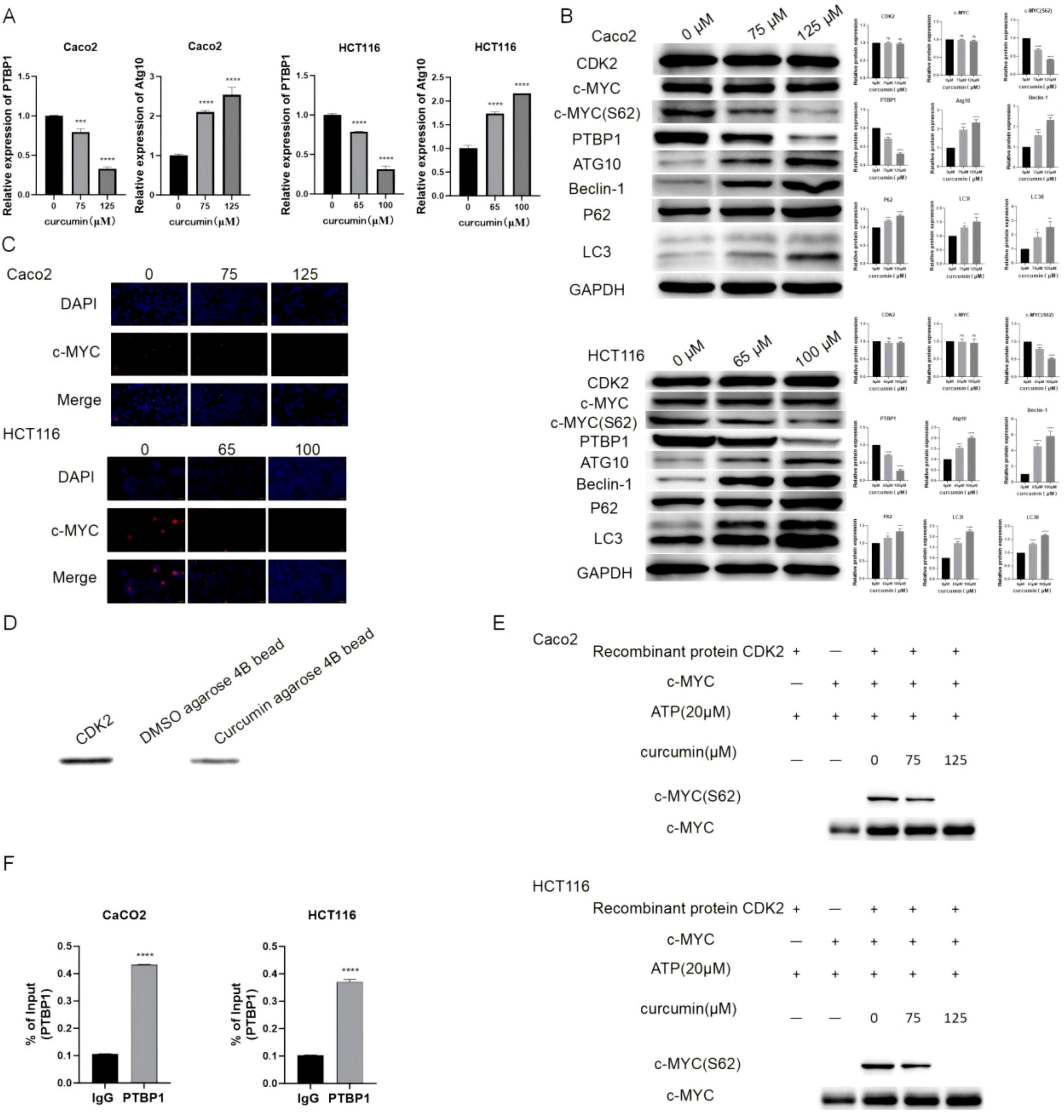
Curcumin modulates key molecular targets in colorectal cancer cells. **(A)** PCR results show that curcumin significantly reduces PTBP1 mRNA levels and increases Atg10 mRNA levels in both CaCo2 and HCT116 cells. **(B)** Western blot analysis demonstrates reduced PTBP1 protein levels and increased autophagy-related proteins (Atg10, Beclin-1, P62, LC3) following curcumin treatment. **(C)** Immunofluorescence analysis shows that curcumin affects the nuclear translocation of c-MYC in both cell lines. **(D)** Pull-down assay confirms direct binding between curcumin and CDK2. **(E)** CDK2 kinase activity assay reveals that curcumin significantly inhibits CDK2 activity. **(F)** ChIP assay shows the binding between c-MYC and PTBP1. ns, p > 0.05; *p < 0.05; **p < 0.01; ***p < 0.001; ****p < 0.0001. Data are presented as mean ± SD, n = 3 for each experiment.

Curcumin treatment significantly reduced PTBP1 mRNA expression while upregulating Atg10 (**Figure 3A**). Western blot analysis revealed decreased PTBP1 protein levels and c-MYC S62 phosphorylation, without affecting total CDK2 or c-MYC expression (**Figure 3B**). Curcumin also enhanced autophagy-related markers including Atg10, Beclin-1, P62, and LC3. Immunofluorescence analysis indicated altered nuclear localization of c-MYC upon curcumin exposure (**Figure 3C**). Direct binding between curcumin and CDK2 was confirmed via pull-down assays (**Figure 3D**), and kinase assays demonstrated dose-dependent CDK2 inhibition (**Figure 3E**). ChIP-qPCR analysis confirmed c-MYC as a transcriptional regulator of PTBP1 in colorectal cancer cells (**Figure 3F**).

CDK2 knockout reproduces the effects of curcumin treatment.

**Figure 4:**
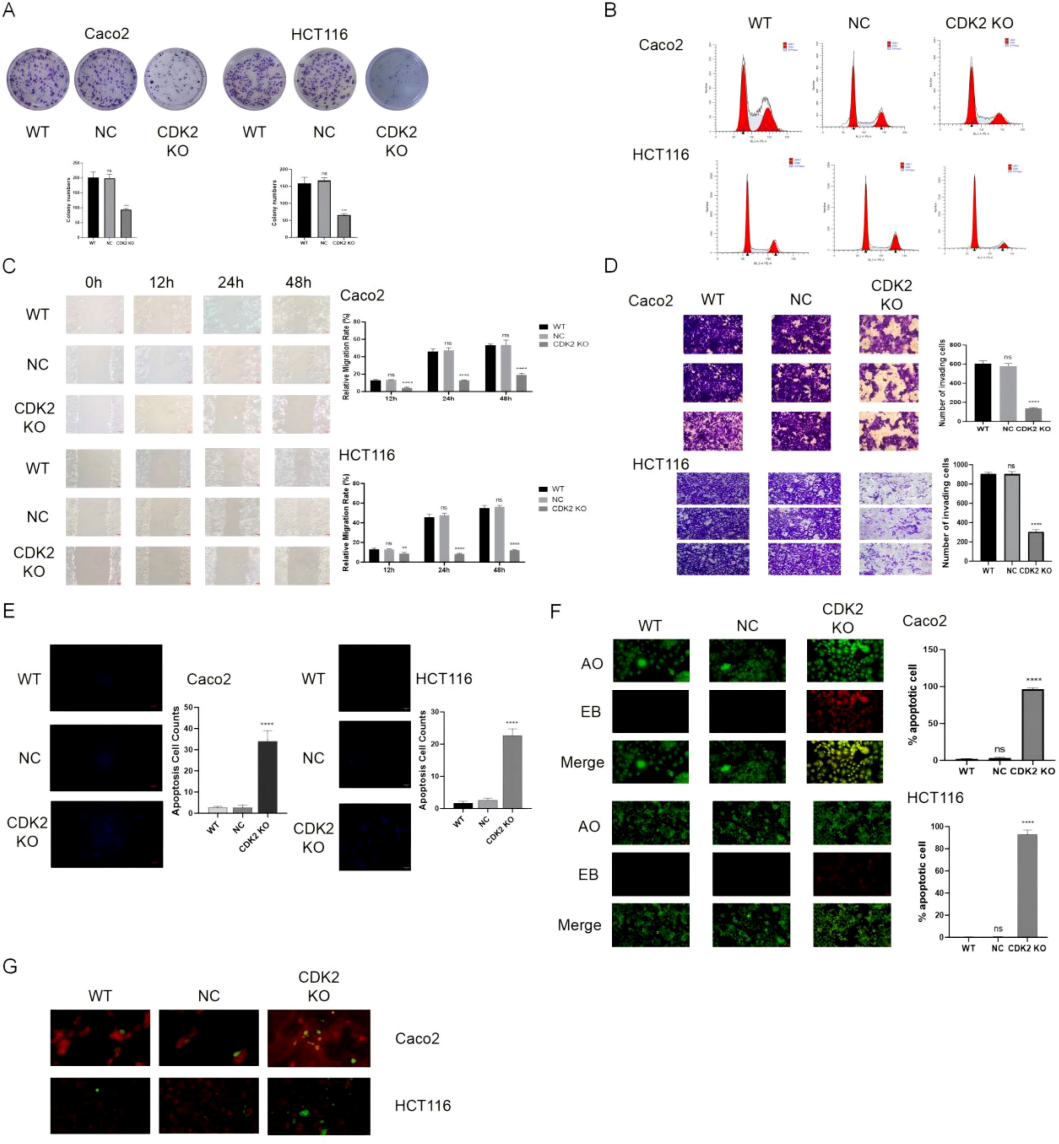
CDK2 knockout reproduces the effects of curcumin treatment. **(A)** Colony formation assays show that CDK2 knockout significantly inhibits cell proliferation in both CaCo2 and HCT116 cell lines. **(B)** Flow cytometry analysis reveals G1 phase arrest in CDK2-deficient cells with corresponding reductions in the S and G2/M phase populations. **(C)** Wound healing assays demonstrate that CDK2 knockout impairs cell migration in both cell lines. **(D)** Transwell invasion assay shows that CDK2 knockout reduces the invasive ability of colorectal cancer cells. **(E)** Hoechst staining shows increased apoptosis in CDK2-deficient cells compared to controls. **(F)** AO staining reveals enhanced autophagic activity in CDK2-knockout cells. **(G)** LC3-mitochondria colocalization shows that CDK2 knockout promotes autophagy in both CaCo2 and HCT116 cells. ns, p > 0.05; **p < 0.01; ***p < 0.001; ****p < 0.0001. Data are presented as mean ± SD, n = 3 for each experiment.

Knocking out CDK2 in colorectal cancer cells significantly reduced their proliferation, as shown by colony formation assays (**Figure 4A**). Flow cytometry results demonstrated G1 phase arrest in CDK2-deficient cells, with corresponding reductions in S and G2/M phase populations (**Figure 4B**). Transwell invasion and wound healing assays showed that CDK2 knockout impaired cell migration and invasion (**Figures 4C, D**). Increased apoptosis rates were observed in CDK2-deficient cells (**Figure 4E**). AO staining and LC3-mitochondria colocalization suggested enhanced autophagic activity in these cells (**Figures 4F, G**).

### Molecular changes associated with CDK2 knockout

**Figure 5:**
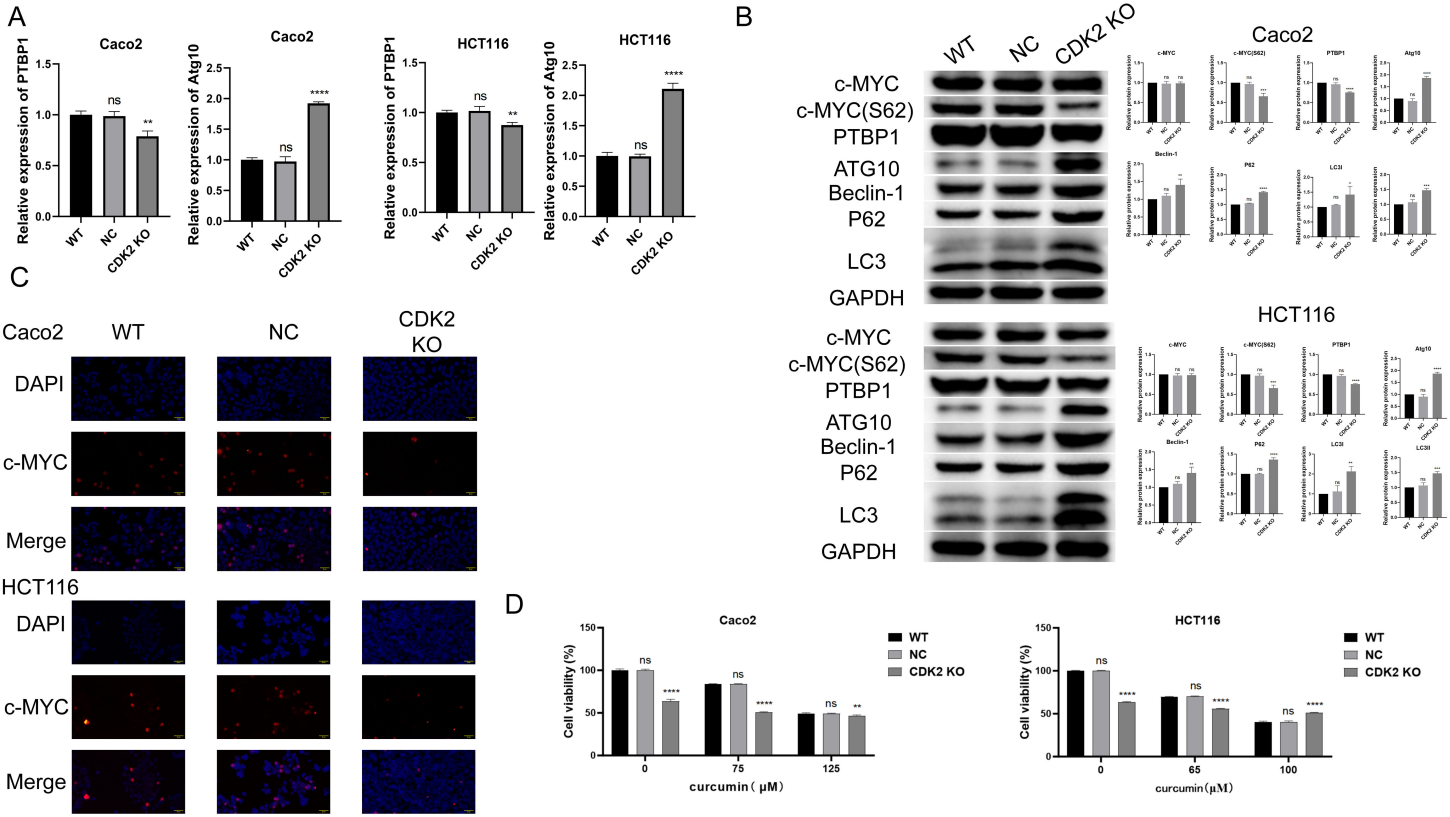
Molecular Changes Associated with CDK2 Knockout. **(A)** PCR results show that CDK2 knockout and curcumin treatment significantly reduce PTBP1 mRNA levels while increasing Atg10 mRNA levels in both cell lines. **(B)** Western blot analysis shows that CDK2 knockout and curcumin treatment reduce PTBP1 protein levels and c-MYC S62 phosphorylation, with an increase in autophagy-related proteins (Atg10, Beclin-1, P62, LC3). **(C)** Immunofluorescence analysis shows altered nuclear localization of c-MYC following CDK2 knockout and curcumin treatment. **(D)** CCK8 assays show that CDK2 knockout reduces cell sensitivity to curcumin treatment in both CaCo2 and HCT116 cells. ns, p > 0.05; *p < 0.05; **p < 0.01; ***p < 0.001; ****p < 0.0001. Data are presented as mean ± SD, n = 3 for each experiment.

CDK2 deletion led to a significant decrease in PTBP1 mRNA levels and an increase in Atg10 mRNA levels, as shown by PCR analysis (**Figure 5A**). Western blot results indicated no significant changes in c-MYC protein expression but showed reduced levels of PTBP1 protein and c-MYC S62 phosphorylation. Additionally, autophagy-related proteins, including Atg10, Beclin-1, P62, and LC3, were upregulated in CDK2-deficient cells (**Figure 5B**). Immunofluorescence analysis confirmed the altered nuclear translocation of c-MYC in the absence of CDK2 (**Figure 5C**). Interestingly, CCK8 assay revealed that CDK2 knockout reduced the sensitivity of colorectal cancer cells to curcumin treatment (**Figure 5D**).

### Curcumin and CDK2 knockout suppress tumor growth *in vivo*

**Figure 6:**
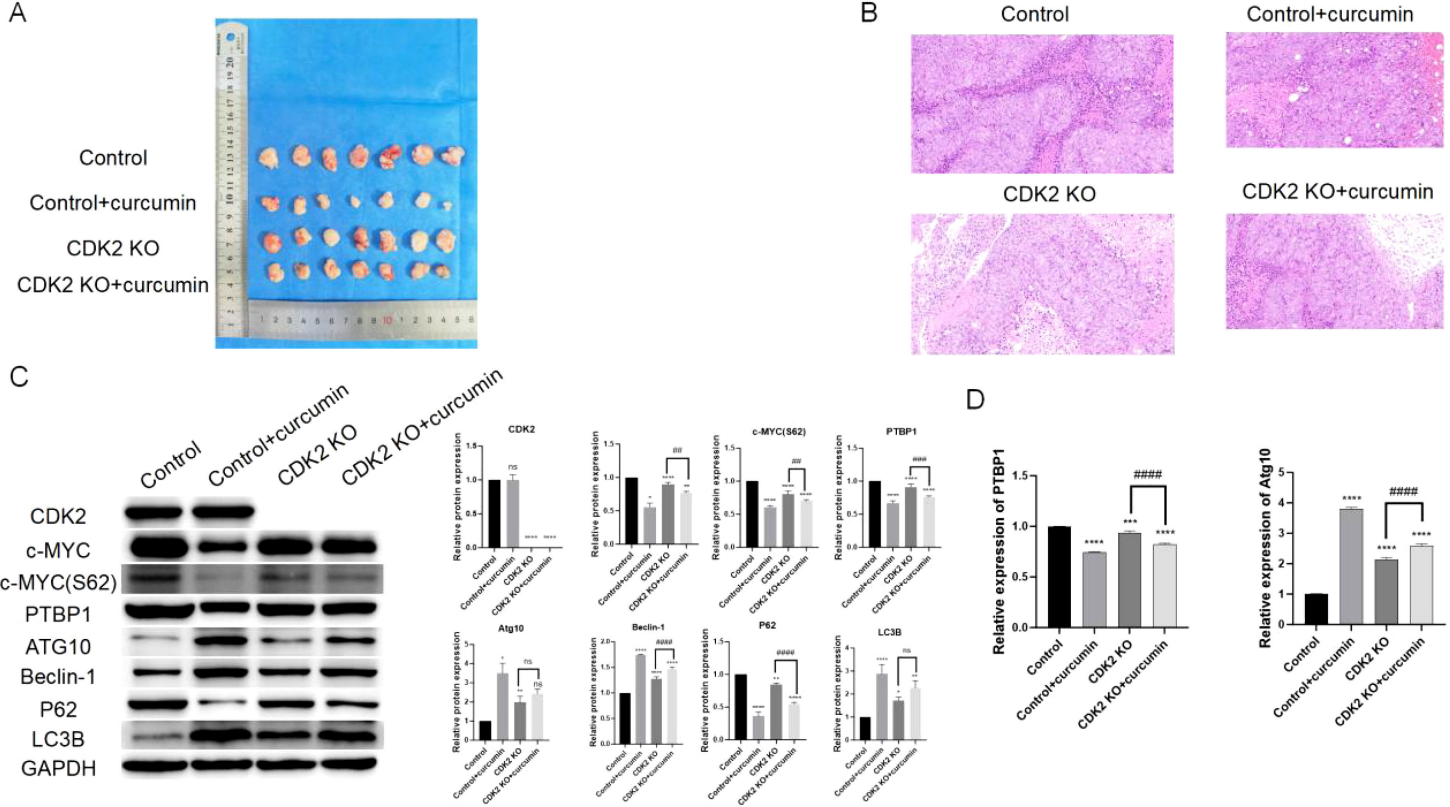
Curcumin and CDK2 knockout suppress tumor growth *in vivo*. **(A)** Xenograft mouse model shows that CDK2 knockout and curcumin treatment significantly reduce tumor volumes. **(B)** H&E staining of tumor tissues reveals reduced tumor cell density, decreased lymphocyte infiltration, and minimized invasive areas in both CDK2 knockout and curcumin-treated groups. **(C)** Western blot analysis of tumor tissues shows reduced PTBP1 protein and c-MYC S62 phosphorylation levels, with an increase in autophagy-related proteins (Atg10, Beclin-1, P62, LC3B). **(D)** PCR analysis confirms that both CDK2 knockout and curcumin treatment reduce PTBP1 mRNA levels while increasing Atg10 mRNA levels in tumor tissues. ns, p > 0.05; *p < 0.05; **p < 0.01; ***p < 0.001; ****p < 0.0001; ##: p < 0.01; ###: p < 0.001; ####; p < 0.0001. Data are presented as mean ± SD, n = 7 for each experiment.

Xenograft mouse models demonstrated that both CDK2 knockout and curcumin treatment significantly reduced tumor volumes (**Figure 6A**). H&E staining revealed that curcumin treatment decreased tumor cell density, reduced lymphocyte infiltration, and minimized invasive areas (**Figure 6B**). Western blot analysis of tumor tissues showed no significant changes in c-MYC protein levels but revealed reduced PTBP1 protein and c-MYC S62 phosphorylation levels, along with increased levels of Atg10, Beclin-1, P62, and LC3B (**Figure 6C**). PCR results further confirmed that both CDK2 knockout and curcumin treatment reduced PTBP1 mRNA levels while increasing Atg10 mRNA levels (**Figure 6D**). Notably, tumors lacking CDK2 exhibited reduced sensitivity to curcumin treatment, as indicated by both Western blot and PCR analyses.

## Discussion

PTBP1 plays a significant role in cancer progression by modulating alternative splicing, RNA stability, and translation. The elevated expression of PTBP1 in colorectal cancer tissues observed in this study supports its oncogenic role, consistent with previous findings that linked PTBP1 to tumor growth and metastasis in multiple cancers (15, 16, 20–24). Our study reveals a novel mechanistic link in which curcumin suppresses PTBP1 expression by targeting the CDK2–c-MYC signaling axis, uncovering a previously uncharacterized regulatory pathway in CRC. Given that PTBP1 has been implicated in autophagy, the observed increase in autophagic activity following curcumin treatment might be partially mediated through PTBP1 downregulation (20, 25–27). Further studies are warranted to dissect the pathways connecting curcumin, PTBP1, and autophagy in colorectal cancer.

Curcumin demonstrated robust anticancer effects in colorectal cancer cells, including suppression of proliferation, migration, and invasion, along with induction of apoptosis and autophagy (28, 29). These phenotypes were accompanied by cell cycle arrest at the G1 phase, suggesting disruption of key oncogenic processes. This broad spectrum of activity highlights curcumin’s potential as a multifaceted therapeutic agent capable of targeting several hallmarks of cancer simultaneously.

Mechanistically, we identified CDK2 as a direct molecular target of curcumin, as validated by pull-down and *in vitro* kinase assays. CDK2, a key regulator of the G1–S transition, is frequently dysregulated in CRC and contributes to tumor progression (17, 18, 30–33). Curcumin-mediated CDK2 inhibition was associated with reduced c-MYC S62 phosphorylation and subsequent PTBP1 downregulation. CDK2 knockout phenocopied many of curcumin’s effects, supporting the central role of CDK2 in mediating its action. Although our ChIP-qPCR results confirmed c-MYC binding to the PTBP1 promoter, the precise transcriptional regulatory mechanism remains to be fully elucidated.

The interplay between curcumin treatment and CDK2 knockout revealed an intriguing dynamic. While both interventions independently suppressed colorectal cancer cell growth and metastasis, CDK2 knockout reduced the cells’ sensitivity to curcumin. This finding suggests that CDK2 may play a central role in mediating curcumin’s anticancer effects. However, curcumin is known to exhibit pleiotropic activity, and additional CDK2-independent pathways—such as NF-κB, PI3K/AKT, and Wnt/β-catenin signaling—may also contribute to its anticancer effects. These parallel or synergistic mechanisms warrant further exploration to fully delineate curcumin’s molecular targets in CRC.

The antitumor efficacy of curcumin was further validated *in vivo*, where it significantly reduced tumor volumes in a xenograft mouse model. Histological analysis confirmed that curcumin decreased tumor cell density and reduced lymphocyte infiltration and invasive areas, consistent with its inhibitory effects on proliferation and metastasis observed *in vitro*. The *in vivo* data highlights its potential for clinical application in colorectal cancer treatment.

Despite these promising findings, several limitations should be acknowledged. First, this study utilized only two CRC cell lines (HCT116 and CaCo2), which may not fully represent the heterogeneity of CRC. Second, no standard chemotherapeutic agents were included as positive controls, limiting direct comparisons with current treatments. Third, while curcumin exhibited potent effects *in vitro*, its clinical translation is hindered by low bioavailability due to poor solubility and rapid systemic clearance. Advanced delivery strategies, including nanoparticle formulations and synthetic analogs, may enhance its pharmacokinetics. Additionally, the mechanistic links between curcumin, PTBP1, and autophagy remain to be clarified. Investigating potential synergistic effects with conventional chemotherapy could further support clinical application. Ultimately, validation in patient-derived organoids and clinical trials will be essential to confirm curcumin’s therapeutic potential in CRC.

## Conclusion

This study demonstrates that curcumin exerts potent anticancer effects in colorectal cancer through a combination of mechanisms, including the inhibition of PTBP1 and CDK2 activity. These findings not only reinforce the therapeutic potential of curcumin but also highlight the importance of targeting key molecular pathways in colorectal cancer progression. Future studies should focus on overcoming the challenges of curcumin delivery and further validating its clinical efficacy in combination with other therapeutic strategies.

## Author note

During the preparation of this manuscript, ChatGPT 4.0 was utilized to assist in language refinement, grammar correction, and improving overall readability. The authors acknowledge the AI tool’s contribution to enhancing the clarity and fluency of the text. However, all final decisions regarding content, structure, and scientific accuracy were made by the authors, who thoroughly reviewed and revised the manuscript to ensure its integrity.

## Data Availability

The original contributions presented in the study are included in the article/supplementary material. Further inquiries can be directed to the corresponding author/s.
