# Correction: Curcumin inhibits colorectal cancer progression by targeting PTBP1 and CDK2-mediated pathways

**DOI:** 10.3389/fonc.2025.1659429

**Published:** 2025-09-02

**Authors:** Hao Zheng, Shenglong Li, Ye Wang, Shuang Su, Yiheng Wang, Fujing Wang

**Affiliations:** Department of General Surgery Ward No.10, Second Affiliated Hospital of Harbin Medical University, Harbin, Heilongjiang, China

**Keywords:** Curcumin, colorectal cancer, PTBP1, CDK2, autophagy the effects of curcumin on crc cell viability, proliferation, migration, invasion

There was a mistake in **Figure 5** as published. In the originally published version, **Figure 5C** was inadvertently duplicated from **Figure 3C**. The corrected **Figure 5** and its caption appear below.

There was a mistake in the caption of **Figure 1** as published. The descriptions for **Figure 1A** and **Figure 1B** were inadvertently reversed. It was mistakenly written as “(A) PCR analysis shows that PTBP1 is significantly overexpressed in colorectal cancer tissues compared to adjacent normal tissues. (B) Western blot analysis confirming the elevated expression of PTBP1 in CRC tissues. Representative images are shown.”. The corrected caption of **Figure 1** appears below.

**Figure 1:**
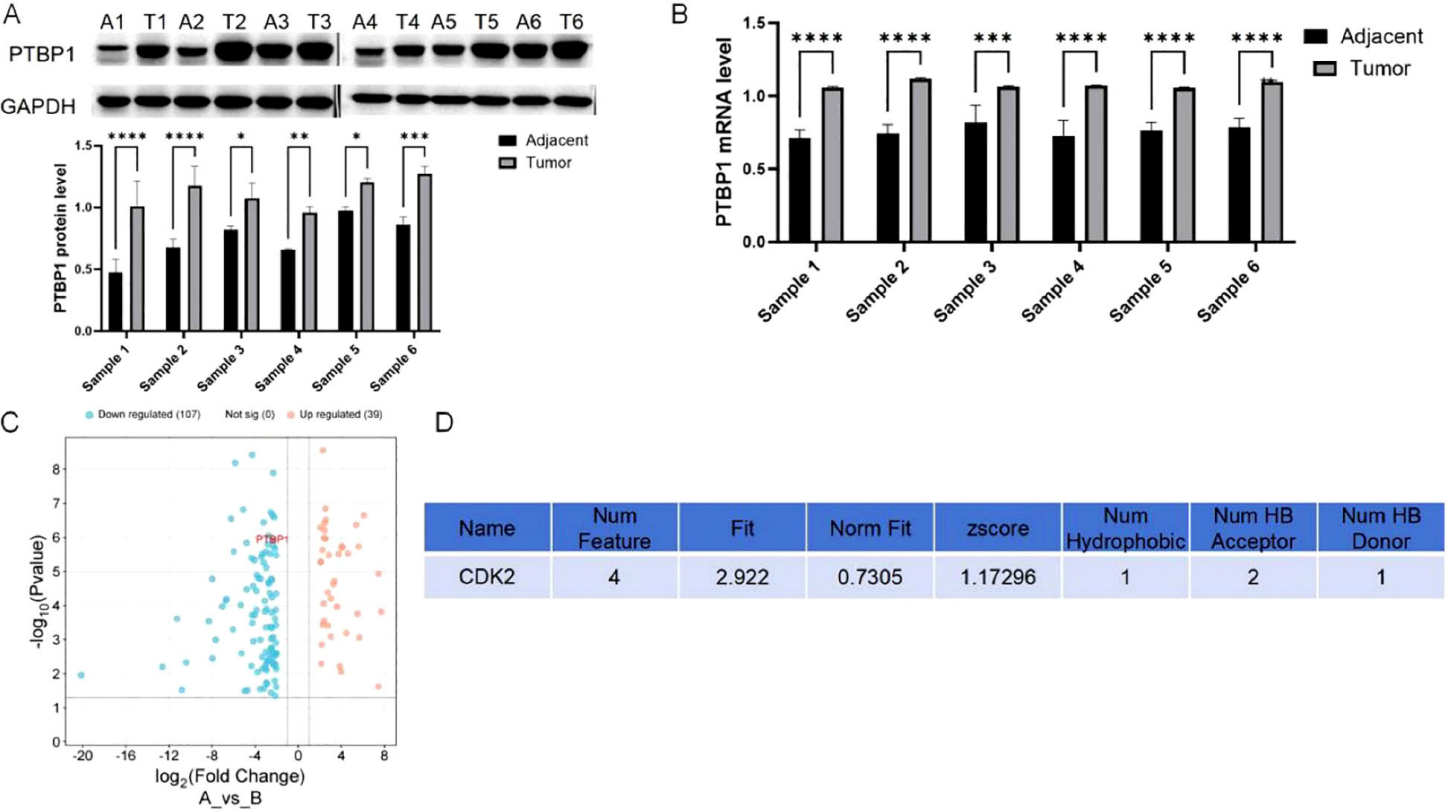
PTBP1 expression is elevated in colorectal cancer and reduced by curcumin. **(A)** Western blot analysis confirming the elevated expression of PTBP1 in CRC tissues. Representative images are shown. **(B)** PCR analysis shows that PTBP1 is significantly overexpressed in colorectal cancer tissues compared to adjacent normal tissues. **(C)** RNA sequencing data from GSE229613 indicates that curcumin treatment significantly reduces PTBP1 expression in colorectal cancer cells. **(D)** Target prediction analysis identifies CDK2 as a potential target of curcumin. Note: ns p > 0.05, * p < 0.05, ** p < 0.01, *** p < 0.001, **** p < 0.0001.

**Figure 5:**
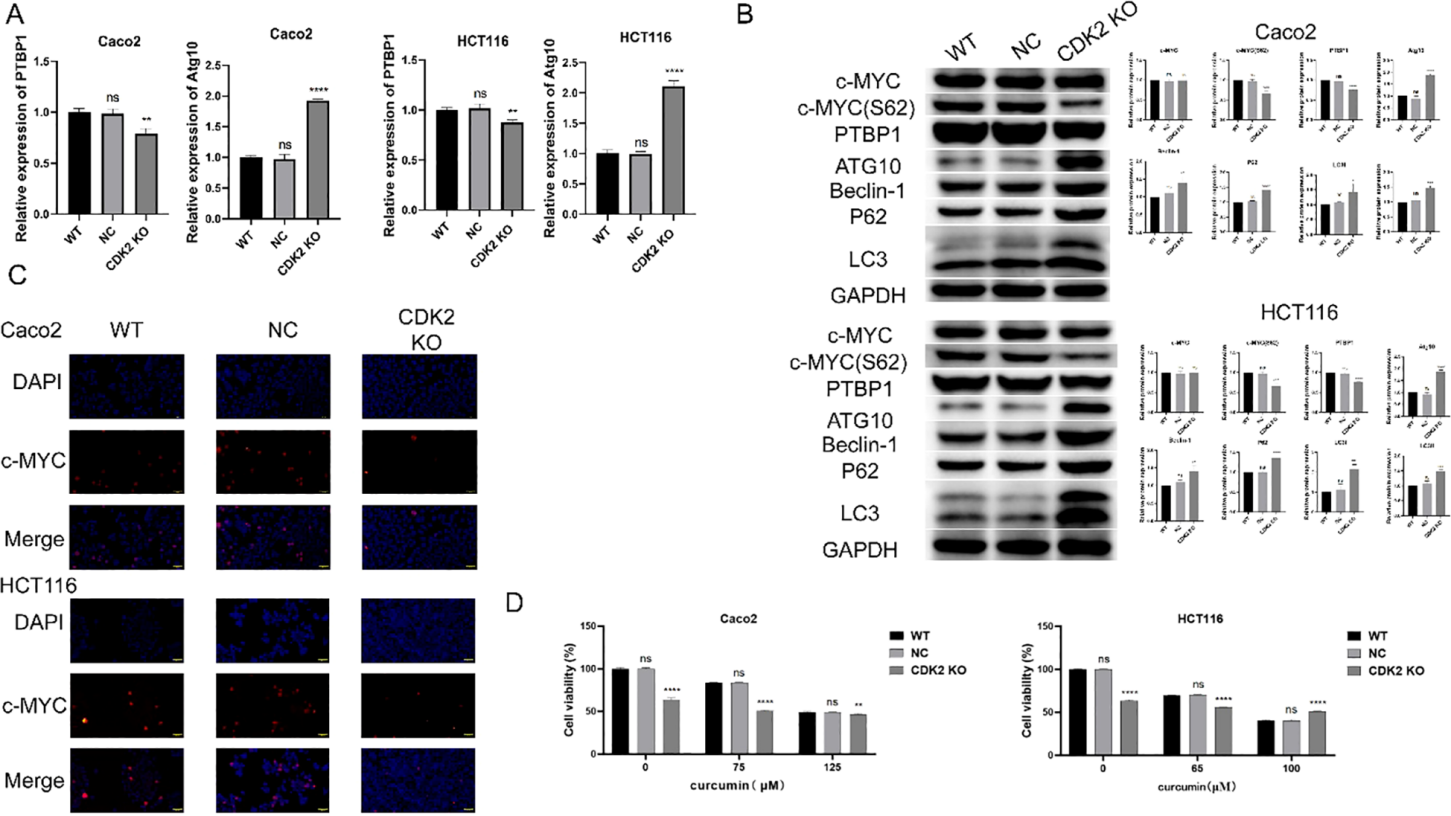
Molecular changes associated with CDK2 knockout. **(A)** PCR results show that CDK2 knockout and curcumin treatment significantly reduce PTBP1 mRNA levels while increasing Atg10 mRNA levels in both cell lines. **(B)** Western blot analysis shows that CDK2 knockout and curcumin treatment reduce PTBP1 protein levels and c-MYC S62 phosphorylation, with an increase in autophagy-related proteins (Atg10, Beclin-1, P62, LC3). **(C)** Immunofluorescence analysis shows altered nuclear localization of c-MYC following CDK2 knockout and curcumin treatment. **(D)** CCK8 assays show that CDK2 knockout reduces cell sensitivity to curcumin treatment in both CaCo2 and HCT116 cells. ns, p > 0.05; *p < 0.05; **p < 0.01; ***p < 0.001; ****p < 0.0001. Data are presented as mean ± SD, n = 3 for each experiment.

“Figure 1. PTBP1 expression is elevated in colorectal cancer and reduced by curcumin. (A) Western blot analysis confirming the elevated expression of PTBP1 in CRC tissues. Representative images are shown. (B) PCR analysis shows that PTBP1 is significantly overexpressed in colorectal cancer tissues compared to adjacent normal tissues. (C) RNA sequencing data from GSE229613 indicates that curcumin treatment significantly reduces PTBP1 expression in colorectal cancer cells. (D) Target prediction analysis identifies CDK2 as a potential target of curcumin. Note: ns p > 0.05, * p < 0.05, ** p < 0.01, *** p < 0.001, **** p < 0.0001.”

In the **Methods** section, we described procedures for immunohistochemistry (IHC); however, no IHC results are presented or discussed in the article. This was an oversight. IHC was originally planned as part of the experimental design, but the data were not included in the final manuscript.

A correction has been made to the section **Methods**, *Immunohistochemistry for Ki67*:

“Paraffin-embedded tumor tissue sections were deparaffinized in xylene and rehydrated through a graded ethanol series. Antigen retrieval was performed by heating the sections in a citrate buffer (pH 6.0) at 95°C for 10 minutes. After cooling, sections were incubated with 3% hydrogen peroxide for 10 minutes to block endogenous peroxidase activity. The sections were then blocked with 5% BSA for 30 minutes at room temperature and incubated overnight at 4°C with a primary antibody against Ki67. After washing, sections were incubated with a secondary antibody conjugated to HRP for 1 hour at room temperature. Color development was achieved using DAB, and sections were counterstained with hematoxylin. After dehydration and clearing, coverslips were mounted, and staining was evaluated under a light microscope. Ki67-positive nuclei were quantified under a light microscope.”

The original version of this article has been updated.

